# Diagnostic accuracy of the gastric cancer T-category with respect to tumor localization

**DOI:** 10.1007/s00423-020-01971-3

**Published:** 2020-08-26

**Authors:** Kenji Nanishi, Katsutoshi Shoda, Takeshi Kubota, Toshiyuki Kosuga, Hirotaka Konishi, Atsushi Shiozaki, Hitoshi Fujiwara, Kazuma Okamoto, Eigo Otsuji

**Affiliations:** Division of Digestive Surgery, Department of Surgery, Kyoto Prefectural University of Medicine, 465 Kawaramachi Hirokoji Kajii-cho, Kamigyo-ku, Kyoto, 602-8566 Japan

**Keywords:** Gastric cancer, Clinical staging, Diagnostic discordance, Tumor localization

## Abstract

**Purpose:**

Diagnosing early gastric cancer (EGC) or advanced gastric cancer (AGC) according to T-category is important for optimal GC treatment; however, the clinical and pathological diagnosis of tumor depths can sometimes vary. This study investigated the accuracy of clinical diagnosis of the tumor depth from the viewpoint of tumor localization and prognosis of patients with GC with discordance between clinical and pathological findings.

**Methods:**

This study enrolled 741 patients with primary GC who underwent curative gastrectomy. Based on the clinical and pathological diagnosis of T-category, the patients were classified into four groups: Early-look EGC, Early-look AGC, Advanced-look EGC, and Advanced-look AGC. Tumor localization was classified longitudinally (the upper [U], middle [M], and lower [L] parts and cross-sectionally (the anterior [Ant] and posterior [Post] walls, and the lesser [Less] and greater [Gre] curvatures).

**Results:**

Of the 462 clinical EGC cases, 52 were Early-look AGC cases that exhibited a significant association of tumor localization with the Post and Less in the U and M locations (UM-PL; *p* = 0.037). An Advanced-look EGC (*p* = 0.031) and Advanced-look AGC (*p* = 0.025) were independent prognostic factors for relapse-free survival each in pathological EGC and AGC, respectively.

**Conclusions:**

Patients with clinically diagnosed EGC but with pathologically diagnosed AGC more frequently presented tumor in the UM-PL than in any other location. Selection of therapeutic strategy according to the clinical diagnosis might be critical; however, it should be also considered that the accuracy of preoperative assessments varies with tumor localization.

**Electronic supplementary material:**

The online version of this article (10.1007/s00423-020-01971-3) contains supplementary material, which is available to authorized users.

## Introduction

Gastric cancer (GC) has been found to be the fifth most common cancer and the third leading cause of cancer-related death worldwide [1]. According to currently available guidelines based on previous studies of GC, the optimal treatment for each patient is determined by finely stratified staging [2, 3]; however, the endoscopic tumor depth may be different from the pathologic tumor depth [4]. Clinical staging of GC has become important for determining the therapeutic strategy. In accordance with a previous study, we established whether it is an early gastric cancer (EGC) only by T-category [5]; moreover, distinguishing EGC from advanced GC (AGC) is particularly meaningful because EGC has the option to undergo endoscopic resection and laparoscopic surgery by clinical staging at many institutions [6–9]. However, clinical EGC (cEGC) is sometimes revealed to be pathological AGC (pAGC) upon examination of the resected specimen, while clinical AGC (cAGC) is often determined to be pathological EGC (pEGC). Regarding the cases in which these discrepancies occur, the detailed clinical features have often not yet been clarified and remain important issues to be solved.

Cross-sectional classification may be as important as longitudinal classification for GC therapy. Previous studies have demonstrated that the expressions of various molecules change depending upon the location in the stomach [10]; therefore, the characteristics of GC may vary according to the tumor location. Other studies have suggested that the prognosis varies depending on the localization of GC and that, in addition to longitudinal classifications (the upper [U], middle [M], and lower [L] parts), cross-sectional classifications (the anterior [Ant] and posterior [Post] walls, and the lesser [Less] and greater [Gre] curvatures) are also associated with long-term survival [11].

The aim of the present study was to investigate the accuracy of the clinical diagnosis of tumor depth from the viewpoint of tumor localization and to evaluate the clinicopathological features and prognosis of GC in patients with discordant clinical and pathological findings.

## Materials and methods

### Patients

Between 2008 and 2017, a total of 917 patients with GC underwent gastrectomy at Kyoto Prefectural University of Medicine Hospital. Patients with residual GC, esophagogastric junction cancer, gastric tube cancer, GC occupying the gastric circumference, multiple GC, administration of neoadjuvant chemotherapy, and re-operation according to the pathological result were excluded from this study. A total of 741 patients with primary GC who underwent curative gastrectomy with D1 plus or D2 lymph node dissection at our university hospital were enrolled in the present retrospective study (Fig. S1).

All included patients had undergone endoscopy and multidetector-row computed tomography (MDCT) before surgery, and the preoperative endoscopic images were reviewed by expert gastrointestinal endoscopists. In our institution, expert gastrointestinal endoscopists classify GC as either EGC or AGC with conventional endoscopy according to the guidelines of the Japan Gastroenterological Endoscopy Society (JGES) as the first step of clinical staging for GC, and if necessary, using endoscopic ultrasound (EUS) or CT as an auxiliary device; subsequently, the invasion to an adjacent organ, lymph node metastasis, and distant metastasis are evaluated by CT and positron-emission tomography/CT (PET/CT). Clinical and pathological staging was performed using the eighth Union for International Cancer Control (UICC) tumor–lymph node–metastasis (TNM) staging classification [12] and the 15th Japanese Classification of Gastric Carcinoma (JCGC) scheme [13]. EGC was defined as invasive carcinoma confined to mucosa and/or submucosa, irrespective of lymph node metastasis [5].

In addition, the study participants were classified, according to T-category, into the following four groups: patients who were clinically diagnosed with cEGC but who were found to have pAGC during the postoperative pathological examination (Early-look AGC), those diagnosed with EGC both clinically and pathologically (Early-look EGC), those clinically diagnosed with cAGC but who were found to have pEGC during the postoperative pathological examination (Advanced-look EGC), and those with AGC diagnosed both clinically and pathologically (Advanced-look AGC). For prognostic analysis, the overall survival (OS) and relapse-free survival (RFS) rates were analyzed in 662 patients, excluding cases with synchronous or metachronous other cancer within 5 years before surgery. Adjuvant chemotherapy was indicated for patients with pathological stage (pStage) II or III (excluded with pT1 and pT3N0), and administration was finally decided considering the patient’s condition including performance status and patient’s expectations. The chemotherapy regimen was basically S-1 single agent for 1 year, and platinum regimens or taxane regimen were added for pStage III if the patient’s condition was tolerable. Follow-up procedures consisting of blood investigations and abdominal ultrasound and CT scans were performed every 3–6 months after surgery. All procedures were performed in accordance with the ethical standards of the responsible committees on human experimentation (institutional and national) and the 1964 Declaration of Helsinki and later versions. Informed consent was obtained from all patients prior to inclusion in this study.

### Definition of sectional location

**Fig. 1:**
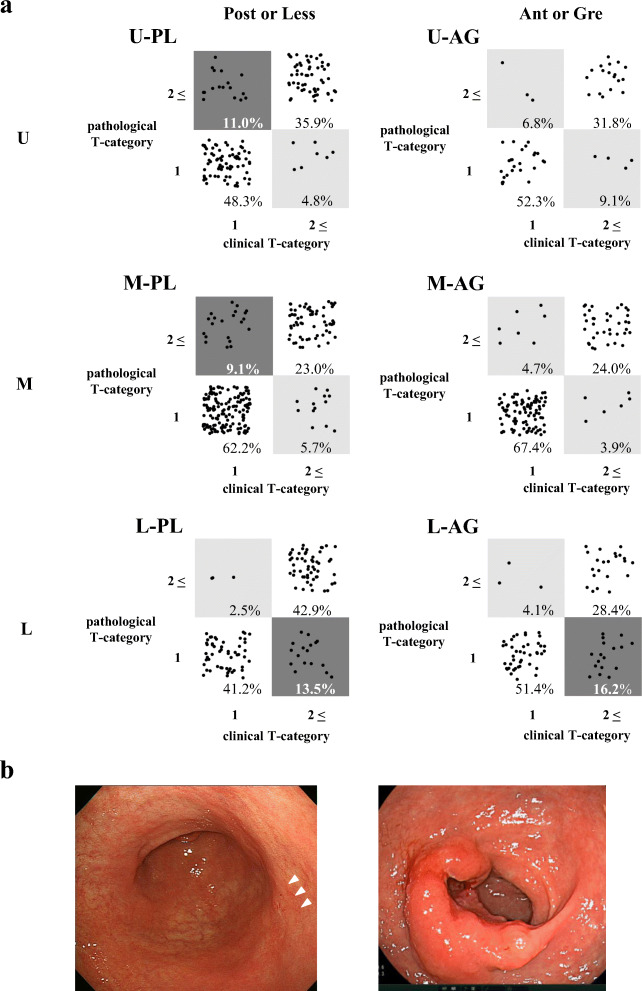
Preoperative diagnostic accuracy according to the tumor location. **a** Scatter plots of the clinical T-category and pathological T-category according to the tumor location are shown. White areas show concordant cases, and gray areas show discordant cases between clinical and pathological diagnoses. A dark gray area indicates a frequency of discordant cases higher than that of a light gray area. **b** Representative endoscopic images of Early-look AGC (left panel) and Advanced-look EGC (right panel)

First, we investigated the clinical features for the longitudinal and cross-sectional tumor locations, respectively. Moreover, tumors were classified into six groups according to the combination of their longitudinal and cross-sectional locations, as follows: Post or Less in the U region (U-PL), Ant or Gre in the U region (U-AG), Post or Less in the M region (M-PL), Ant or Gre in the M region (M-AG), Post or Less in the L region (L-PL), and Ant or Gre in the L region (L-AG) (Fig. 1).

### Histological evaluation

In accordance with the 15th JCGC scheme [13], the primary tumors were cut crosswise through the center of the tumor. Tumors were sectioned in their entirety parallel to the reference line at 5-mm intervals. The resected specimens were fixed in 10% buffered formalin solution, embedded in paraffin, and stained with hematoxylin and eosin. The clinicopathological features of all study participants were obtained from their hospital records based on the eighth UICC TNM classification [12] and the 15th JCGC scheme [13].

### Statistical analysis

Fisher’s exact probability test and the chi-squared test were performed to compare categorical variables between the two groups, while non-parametric tests were used for subgroup comparisons. Multivariate analysis was performed using multinomial logistic regression to examine clinicopathologic factors affecting to endoscopic diagnosis. OS and RFS rates were calculated via Kaplan–Meier analysis, with the date of gastrectomy designated as the starting point. Differences in survival rates were examined by log-rank test. The Cox proportional hazards models were used to estimate potential clinicopathologic characteristics affecting prognosis. All statistical analysis was performed using JMP version 13 (ASA Institute, Cary, NC, USA). All statistical tests except for the paired tests were two-sided. Statistical significance was accepted at *p* < 0.05.

## Results

### Clinicopathological characteristics

This study evaluated 741 patients who met all of the above-defined criteria, with 410, 52, 57, and 222 patients categorized into the Early-look EGC, Early-look AGC, Advanced-look EGC, and Advanced-look AGC groups, respectively (Table 1). Age (*p* < 0.001), tumor longitudinal localization (*p* < 0.001), tumor size (*p* < 0.001), macroscopic appearance (*p* < 0.001), surgical approach (*p* < 0.001), surgical procedure (*p* < 0.001), lymph node dissection (*p* < 0.001), and adjuvant chemotherapy (*p* < 0.001) were significantly different among above four groups.

**Table 1:** Correlation between clinicopathological characteristics and endoscopic diagnosis of tumor depth for GC

Variables	Early-look EGC^a^ (%) *n* = 410	Early-look AGC^a^ (%) *n* = 52	Advanced-look EGC^a^ (%) *n* = 57	Advanced-look AGC^a^ (%) *n* = 222	*p* value
Age					
Mean ± SD (years)	65.3 ± 0.5	65.5 ± 1.7	67.7 ± 1.5	68.8 ± 0.8	*< 0.001*
Sex					
M	268 (65.4%)	39 (75.0%)	38 (66.7%)	161 (72.5%)	0.203
F	142 (34.6%)	13 (25.0%)	19 (33.3%)	61 (27.5%)	
BMI					
Mean ± SD (kg/m^2^)	22.8 ± 3.5	21.9 ± 3.5	23.0 ± 3.8	22.2 ± 3.2	0.104
Histopathology^b^ (biopsy)					
Intestinal type	223 (55.2%)	22 (44.0%)	33 (58.9%)	128 (60.1%)	0.241
Diffuse type	181 (44.8%)	28 (56.0%)	23 (41.1%)	85 (39.9%)	
Unknown	6	2	1	9	
Tumor localization 1					
U	93 (22.7%)	19 (36.5%)	11 (19.3%)	66 (29.7%)	*< 0.001*
M	230 (56.1%)	27 (51.9%)	18 (31.6%)	84 (37.8%)	
L	87 (21.2%)	6 (11.5%)	28 (49.1%)	72 (32.4%)	
Tumor localization 2					
Post	113 (27.6%)	14 (26.9%)	18 (31.6%)	54 (24.3%)	0.247
Less	149 (36.3%)	26 (50.0%)	18 (31.6%)	102 (45.9%)	
Ant	69 (16.8%)	7 (13.5%)	9 (15.8%)	35 (15.8%)	
Gre	79 (19.3%)	5 (9.6%)	12 (21.1%)	31 (14.0%)	
Tumor size (mm)					
≥ 20	289 (71.2%)	49 (94.2%)	50 (87.7%)	220 (99.1%)	*< 0.001*
< 20	117 (28.8%)	3 (5.8%)	7 (12.3%)	2 (0.9%)	
Unknown	4	0	0	0	
Macroscopic appearance					
Localized	16 (3.9%)	3 (5.8%)	20 (35.1%)	87 (39.2%)	*< 0.001*
Diffuse	394 (96.1%)	49 (94.2%)	37 (64.9%)	135 (60.8%)	
Surgical approach					
Open	48 (11.7%)	11 (21.2%)	40 (70.2%)	199 (89.6%)	*< 0.001*
Laparoscopic	362 (88.3%)	41 (78.8%)	17 (29.8%)	23 (10.4%)	
Surgical procedure					
TG	66 (16.1%)	15 (28.8%	18 (31.6%)	101 (45.5%)	*< 0.001*
DG	293 (71.5%)	31 (59.6%)	38 (66.7%)	116 (52.3%)	
Others	51 (12.4%)	6 (11.5%)	1 (1.8%)	5 (2.3%)	
Lymph node dissection					
< D2	387 (94.4%)	47 (90.4%)	17 (29.8%)	52 (23.4%)	*< 0.001*
≥ D2	23 (5.6%)	5 (9.6%)	40 (70.2%)	170 (76.6%)	
Adjuvant chemotherapy					
(+)	13 (3.2%)	14 (27.5%)	5 (8.9%)	103 (47.9%)	*< 0.001*
S-1	12	12	4	83	
S-1 + platinum	0	1	1	13	
Others	1	1	0	7	
(−)	396 (96.8%)	37 (72.5%)	51 (91.1%)	112 (52.1%)	
Unknown	1	1	1	7	

^a^Disease stage was defined in accordance with the International Union Against Cancer 7th tumor-lymph node-metastases classification using surgical-pathological findings

^b^According to Lauren classification using most predominant histopathological finding

*EGC*, early gastric cancer; *AGC*, advanced gastric cancer; *SD*, standard deviation; *BMI*, body mass index; *TG*, total gastrectomy; *DG*, distal gastrectomy

Italic entries show *p* value <0.05

### Predictable factors related to discordance between clinical and pathological findings

Although no significant differences were found when comparing the cross-sectional locations identified among the four groups (Post, Less, Ant, and Gre), we did recognize a tendency for more frequent occurrences of Early-look AGC in the PL regions (*p* = 0.065). Scatterplots of the clinical and pathological diagnoses in relation to the six locations are shown (Fig. 1). Early-look AGC was found in 11.0% in the U-PL and 9.1% in the M-PL, which were higher frequencies than those of the other locations. Moreover, the U-PL and M-PL (UM-PL) areas were more correlated with Early-look AGC cases than the other areas among the cEGC patients (*p* = 0.012). Separately, in the cAGC patients, the L area was significantly correlated with Advanced-look EGC cases in comparison with the other areas (*p* = 0.035).

Of the 462 cEGC patients, 52 cases had pAGC (Early-look AGC); these patients showed a significant association with tumor localization in the UM-PL (*p =* 0.037) and a larger tumor size (*p <* 0.001) in the multivariate analysis (Table 2). Conversely, among the 279 cAGC patients, 57 cases were included in the Advanced-look EGC group, and these cases showed a significant association with tumor localization in the L region (*p =* 0.021) and a smaller tumor size (*p =* 0.016) (Table S1).

**Table 2:** Clinicopathological risk factors for diagnosing pAGC as cEGC

Variables	pEGC (%) *n* = 410	pAGC (%) *n* = 52	Univariate analysis	Multivariate analysis		
				Odds ratio	*p* value	95% CI
Age						
< 65	173 (42.2%)	24 (46.2%)	0.587			
≥ 65	237 (57.8%)	28 (53.8%)				
Sex						
M	268 (65.4%)	39 (75.0%)	0.212			
F	142 (34.6%)	13 (25.0%)				
BMI						
≥ 22	237 (57.9%)	26 (50.0%)	0.300			
< 22	172 (42.1%)	26 (50.0%)				
Histopathology^a^ (biopsy)						
Intestinal type	223 (55.2%)	22 (44.0%)	0.175			
Diffuse type	181 (44.8%)	28 (56.0%)				
Unknown	6	2				
Tumor localization						
UM-PL	213 (52.0%)	37 (71.2%)	*0.012*	*2.18*	*0.037*	*1.04–4.54*
Other	197 (48.0%)	15 (28.8%)				
Tumor size (mm)						
< 20	289 (71.2%)	49 (94.2%)	*< 0.001*	*4.68*	*0.014*	*1.37–16.02*
≥ 20	117 (28.8%)	3 (5.8%)				
Macroscopic appearance						
Localized	16 (3.9%)	3 (5.8%)	0.461			
Diffuse	394 (96.1%)	49 (94.2%)				
Surgical approach						
Open	48 (11.7%)	11 (21.2%)	0.055			
Laparoscopic	362 (88.3%)	41 (78.8%)				
Surgical procedure						
TG	66 (18.4%)	15 (32.6%)	0.023	*1.56*	*0.243*	*0.74–3.28*
DG	293 (81.6%)	31 (67.4%)				
Others	51	6				
Lymph node dissection						
< D2	387 (94.4%)	47 (90.4%)	0.254			
≥ D2	23 (5.6%)	5 (9.6%)				
Adjuvant chemotherapy						
(+)	13 (3.2%)	14 (27.5%)	*< 0.001*	8.85	*< 0.001*	*3.51–22.29*
(−)	396 (96.8%)	37 (72.5%)				
Unknown	1	1				

^a^According to Lauren classification using most predominant histopathological finding

*cEGC*, clinical early gastric cancer; *pEGC*, pathological early gastric cancer; *pAGC*, pathological advanced gastric cancer; *CI*, confident interval; *BMI*, body mass index; *TG*, total gastrectomy; *DG*, distal gastrectomy

Italic entries show *p* value <0.05

### Clinical outcomes after surgery

**Fig. 2:**
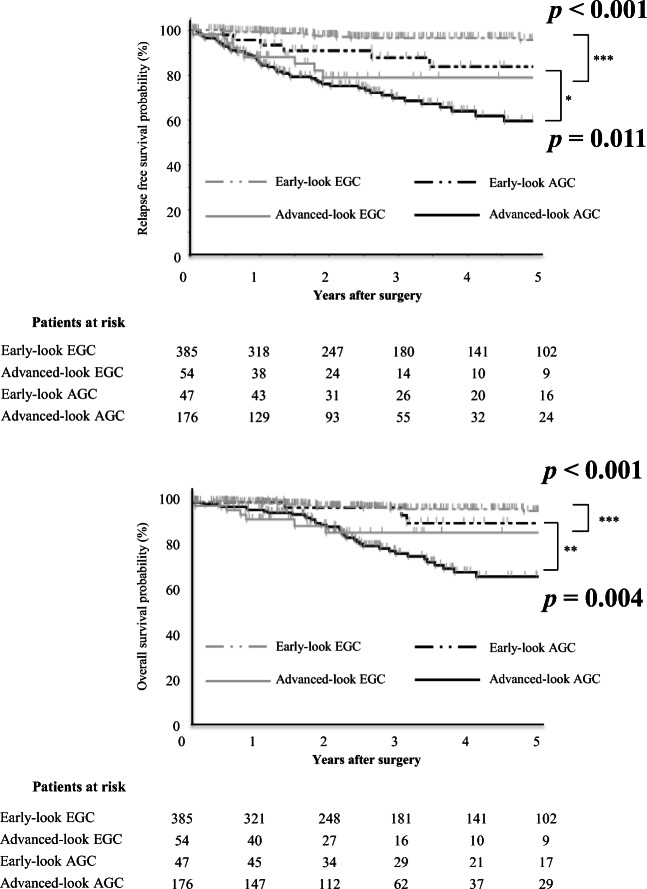
Prognostic factors according to the pre- and postoperative T-categories in patients with GC. Kaplan–Meier curves for the RFS (top) and OS (bottom) rates of GC patients according to the pre- and postoperative T-categories

Figure 2 shows the prognostic impact of the preoperative endoscopic appearance in patients with GC. The analysis results divided into four groups (Early-look EGC, Advanced-look EGC, Early-look AGC, and Advanced-look AGC) revealed that the prognosis was significantly stratified for both OS (*p* < 0.001) and RFS (*p* < 0.001). When comparing the prognosis of patients with cEGC and cAGC according to the pT-category (Early-look vs. Advanced-look EGC, Early-look vs. Advanced-look AGC) to focus on the impact of cT-category on the prognosis, we found that patients with cEGC on clinical estimation had a significantly better prognosis than patients with cAGC on clinical estimation in both comparisons. Among patients with pAGC on the pathological examination, the Early-look AGC cases showed significantly higher OS and RFS rates (*p* = 0.004 and *p* = 0.011, respectively) than the Advanced-look AGC cases. The tendency was similar in the subgroup analysis conducted for each stage (pStage I/II/III), although no significant difference was detected because of the limited number of cases. In the subgroup analysis, we analyzed only pEGC in pStage I and pAGC in pStage II/III, because of the uneven number of patients (Fig. S2). We then analyzed clinicopathological characteristics affecting prognosis in patients with pAGC. The Cox proportional hazards model revealed that low body mass index (RFS, *p* = 0.030; OS, *p* = 0.021), total gastrectomy (RFS, *p* = 0.004; OS, *p* = 0.024), D1 plus lymph node dissection (RFS, *p* = 0.044; OS, *p* = 0.039), pathological node-positive (RFS, *p* = 0.005; OS, *p* = 0.008), and cAGC (RFS, *p* = 0.007; OS, *p* = 0.017) were independent prognostic factors for RFS and OS in patients with pAGC (Table 3). As only a few patients with pAGC had < 20 mm tumor and few of them underwent laparoscopic gastrectomy with >D2 lymph node dissection, the tumor size and the surgical approach were excluded from the variables of multivariate analysis to maintain statistically reliability.

**Table 3:** Multivariate analysis for clinicopathological characteristics affecting prognosis in patients with pAGC

Variables	RFS			OS		
	HR	95% CI	*p* value	HR	95% CI	*p* value
Age ≥ 65 (vs. < 65)	0.81	0.43–1.57	0.520	0.92	0.44–2.00	0.829
Female (vs. male)	1.24	0.582.51	0.564	1.37	0.59–3.03	0.456
BMI < 22 (vs. ≥ 22)	2.01	1.07–3.94	0.030	2.40	1.14–5.28	0.021
cAGC (vs. cEGC)	*4.85*	*1.49–22.16*	*0.007*	*5.45*	*1.32–37.77*	*0.017*
Macroscopic diffuse appearance (vs. localized appearance)	0.62	0.32–1.20	0.156	0.51	0.24–4.17	0.077
TG (vs. DG)	*2.54*	*1.36–4.87*	*0.004*	*2.28*	*1.12–4.75*	*0.024*
Lymph node dissection < D2 (vs. ≥ D2)	*2.25*	*1.02–4.75*	*0.044*	*2.54*	*1.05–5.87*	*0.039*
Histopathological intestinal type (vs. diffuse type)^a^	1.38	0.68–2.83	0.369	1.13	0.49–2.62	0.774
pN stage pN1–3 (vs. pN0)	*2.54*	*1.32–5.27*	*0.005*	*2.81*	*1.31–6.62*	*0.008*
Lymphatic invasion (vs. negative)	1.41	0.67–3.26	0.379	0.94	0.42–2.34	0.890
Venous invasion (vs. negative)	0.89	0.47–1.74	0.243	1.03	0.48–2.29	0.934
Adjuvant chemotherapy (vs. absence)	1.47	0.77–2.88	0.156	1.20	0.56–2.62	0.634

^a^According to Lauren classification using most predominant histopathological finding

*pAGC*, pathological advanced gastric cancer; *cAGC*, clinical advanced gastric cancer; *cEGC*; clinical early gastric cancer; *RFS*, relapse-free survival; *OS*, overall survival; *HR*, hazard ratio; *CI*, confident interval; *BMI*, body mass index; *TG*, total gastrectomy; *DG*, distal gastrectomy

Italic entries show *p* value <0.05

Among the patients with pEGC, the Advanced-look EGC cases exhibited a significantly poorer prognosis in terms of both OS and RFS (*p* < 0.001 and *p* < 0.001, respectively). In these cases, the Cox proportional hazards model showed that older age (RFS, *p* = 0.018; OS, *p* = 0.015), D1 plus lymph node dissection (RFS, *p* = 0.016; OS, *p* = 0.024), and adjuvant chemotherapy (RFS, *p* = 0.016; OS, *p* = 0.013) were independent prognostic factors for RFS and OS. Advanced-look EGC case designation for RFS (*p* = 0.016) and macroscopic diffuse appearance for OS (*p* = 0.039) were the independent prognostic factors (Table S2). Concerning the recurrence pattern, there were no significant differences in the comparison between the Advanced-look EGC and Early-look EGC cases.

## Discussion

To determine the appropriate treatment for GC, ensuring an accurate preoperative assessment of the tumor depth has become increasingly important due to the development of minimally invasive treatment modalities such as endoscopic resection and laparoscopic surgery. Japanese gastric cancer guidelines determine the indication of treatment according to cT-category. For endoscopic resection, the absolute indication [14, 15] is either differentiated type (approximately equal intestinal type) cT1a tumor without ulcer or differentiated type (approximately equal intestinal type) cT1a (≤ 3 cm) tumor with ulcer, and the expanded indication [16] is undifferentiated type (approximately equal diffuse type) cT1a (≤ 2 cm) tumor without ulcer. Furthermore, the lesion out of indication for endoscopic resection is subjected to surgical resection. In Japan, many institutions employ laparoscopic surgery, as it is minimally invasive, for surgical resection for cT1 lesion. For lesions endoscopically resected, the degree of cure is determined by the pathological diagnosis after endoscopic resection and the subsequent treatment policy (including laparoscopic gastrectomy) is determined. This flow of indications is based on endoscopic T-category, biopsy histology, and endoscopically resected tumor histology rather than the clinical stage. Thus, endoscopic determination of T-category may be of great clinical significance.

The introduction of MDCT and endoscopic ultrasonography (EUS) has increased the accuracy of the clinical diagnosis and has had an effect on therapy selection [17, 18]; in particular, EUS is recommended in the National Comprehensive Cancer Network guidelines [2]. Although EUS is a moderately accurate imaging modality in terms of elucidating tumor depth in GC [19], conventional endoscopy also enables a distinction to be made between EGC and AGC. Moreover, EUS is not available in all institutions, and the sensitivity and specificity of EUS have been reported to range widely [20, 21]. Further, some previous studies have suggested that conventional endoscopy could achieve a similar degree of accuracy than that of EUS [22–24], and were conducted using conventional endoscopy in an effort to differentiate between EGC and AGC [25]. Therefore, the guidelines of the JGES have suggested that the T-category diagnosis is more generally performed by conventional endoscopy and proposed to use EUS in a supplementary fashion to diagnose whether the tumor is T1a or T1b [26]. In our institution, we first classify GC into EGC or AGC with conventional endoscopy and then perform EUS or high-magnification endoscopy for further assessment, if necessary, according to the above guidelines. Furthermore, we used CT or PET/CT for preoperative diagnosis to evaluate serosal invasion or invasion of adjacent organ, N factor, and M factor. EUS for gastric cancer was performed in 27 cases (cEGC: 22 cases; cAGC: 5 cases) in our institution during the same period as this study. There was no case with a different T-category between the conventional endoscopy and EUS, and only 2 of 22 cases diagnosed as cEGC were Early-look AGC (pT2).

Regarding tumor site localization, the nature of the GC tumor has been reported to differ depending on its location [27]. Lee et al. suggested that a longitudinal location might affect the accuracy of the endoscopic examination [25]. Recently, the importance of evaluating not only the longitudinal location but also the cross-sectional location has been suggested [11]. However, few studies to date have focused on the cross-sectional location; Jung et al. examined the relationship between the cross-sectional location and clinicopathological features, but only for EGC [11]. Therefore, we integrated both longitudinal and cross-sectional localizations of GC. To our knowledge, the present study is the first report to examine in detail the relationship between longitudinal and cross-sectional locations and the accuracy of clinical diagnosis of tumor depth in patients with GC. When longitudinal localization was stratified according to the UM and L regions in cAGC, we noted significantly more frequent occurrences of Advanced-look EGC cases in the latter (*p* = 0.021), while, in cEGC, we noted a tendency for more frequent occurrences of Early-look AGC cases in the former (*p* = 0.067). In addition, although no significant difference was found when comparing the four cross-sectional location groups, we did find a tendency for more frequent occurrences of Early-look AGC cases in PL regions among cEGC patients (*p* = 0.065). Therefore, in the present study, we focused on the L region in the context of cAGC and the UM-PL region in the context of cEGC, respectively. In the cEGC group, a UM-PL region was chosen as a predictor for an Early-look AGC case status in the multivariate analysis. The reason for underdiagnosis in the UM-PL region was not clear; however, we did not observe significant clinicopathological characteristics of the tumor despite the high frequency of Early-look AGC cases in this region. These results suggest that the biological characteristics of cancer may differ depending on the site affected in the stomach. Among the cAGC cases, we found that an L region was significantly associated with an Advanced-look EGC status. This region is known to be associated with both peptic ulcers and ulcerated GC. This association may explain the overdiagnosis by preoperative endoscopic assessment in cases with a coexisting peptic ulcer in the L region [28]. Over-staging also harbors a risk for the patient; surgical over-treatment can lead to higher postoperative dysfunction or complication rates. Therefore, a strategy is required to improve the diagnostic accuracy of endoscopy, such as a repeated preoperative endoscopic assessment after the administration of medical treatment for a peptic ulcer in an L-region location.

We next investigated the prognostic impact of clinical and pathological evaluations. Clinicians are often unsure of the adequacy of the extent of surgical dissection and adjuvant chemotherapy in Early-look AGC and Advanced-look EGC cases, respectively. In the present study, the Early-look AGC cases showed a significantly better prognosis than did the Advanced-look AGC cases in terms of both OS and RFS among pathologically diagnosed AGC patients. Moreover, it was extremely interesting to note that Early-look AGC in fact exhibited better prognosis than Advanced-look EGC cases. The comparison of clinicopathological factors between Early-look AGC and Advanced-look EGC cases is shown in Table S1. In Early-look AGC cases, the tumor was more often located in the UM-PL region (*p* < 0.001) and laparoscopic surgery (*p* < 0.001), <D2 lymph node dissection (*p* < 0.001), and adjuvant chemotherapy (*p* = 0.012) were more often performed than those in Advanced-look EGC cases. Although Early-look AGC cases would be supposed to exhibit worse prognosis than Advanced-look EGC cases since pN positive rate was not significantly different between them, the actual result was the opposite. Perhaps, laparoscopic gastrectomy with D1+ lymph node dissection could be a reasonable strategy for Early-look AGC cases, and rather the absence of adjuvant chemotherapy for Advanced-look EGC cases may need to be reduced. Some previous studies suggested that D1 plus dissection was recommended for patients with an Early-look AGC status, assuming that no prognostic differences existed between D1 plus and D2 dissection in these cases [29, 30]. Our presented results may also support this opinion. For EGC on pathologic examination, Kitamura et al. suggested that Advanced-look EGC cases should be treated as GC with invasion extending into the proper muscle layer [31]. Supportively to this previous report, our study demonstrated that an Advanced-look EGC status was also an independent prognostic factor for RFS of pEGC. cAGC appears to be a critical biological factor in patients with pEGC (Advanced-look EGC), and patients with this diagnosis might be considered for adjuvant chemotherapy a high-risk group for pEGC. Therefore, further research is necessary to ensure the adequacy of D1 plus dissection for Early-look-AGC cases or adjuvant chemotherapy for Advanced-look EGC cases.

The present study had some limitations that must be mentioned. First, it was limited by a small sample size, which prevented us from drawing more concrete conclusions regarding the accuracy of the tumor localization assessment and determination of the therapeutic strategy based on the preoperative assessment. Further studies involving a larger number of patients are warranted. However, the present study did demonstrate the clinical potential of clinical staging of tumor depth.

## Conclusion

The accuracy of clinical assessments varies with tumor localization in GC. Clinical diagnosis of EGC or AGC is likely to have a strong impact on patient prognosis based on the choice of therapeutic modalities. Therefore, clinician needs to make a strategy in consideration of possible overstaging or understaging depending on tumor localization. Further research is needed to clarify which diagnosis should be emphasized to decide the treatment when clinical and pathological T-categories are different.

## Electronic supplementary material

Fig. S1 A flow chart of patient selection. Patients with residual GC, esophagogastric junction cancer, gastric tube cancer, GC occupying the gastric circumference, multiple GC, administration of neoadjuvant chemotherapy and re-operation according to pathological result were excluded from 917 patients with GC underwent gastrectomy. A total of 741 patients were enrolled in the present retrospective study and after excluding patients with synchronous or metachronous other cancer within 5 years before surgery, prognosis of 662 patients were analyzed (PPTX 73 kb).

Fig. S2 Relation between clinical T-category and survival curve (OS and RFS) by each pStage. Kaplan–Meier curves for the RFS (left) and OS (right) rates of GC patients by each pStage. (PPTX 328 kb).

Fig. S3 Survival curve by prognostic factors in patients with pEGC. Kaplan–Meier curves for the RFS rates of GC patients by prognostic factors in patients with pEGC. (PPTX 208 kb).

ESM 1 (DOCX 146 kb).
