# Supplementary material for: Diagnostic accuracy of the gastric cancer T-category with respect to tumor localization
Source: Langenbecks Arch Surg. 2020 Aug 26;405(6):787–96. doi: 10.1007/s00423-020-01971-3 (PMC7471143; doi:10.1007/s00423-020-01971-3)
Supplement: Supplementary file 1 — A flow chart of patient selection. Patients with residual GC, esophagogastric junction cancer, gastric tube cancer, GC occupying the gastric circumference, multiple GC, administration of neoadjuvant chemotherapy and re-operation according to pathological result were excluded from 917 patients with GC underwent gastrectomy. A total of 741 patients were enrolled in the present retrospective study and after excluding patients with synchronous or metachronous other cancer within 5 years before surgery, prognosis of 662 patients were analyzed (PPTX 73 kb). [file 423_2020_1971_MOESM1_ESM.pptx]

## Slide 1
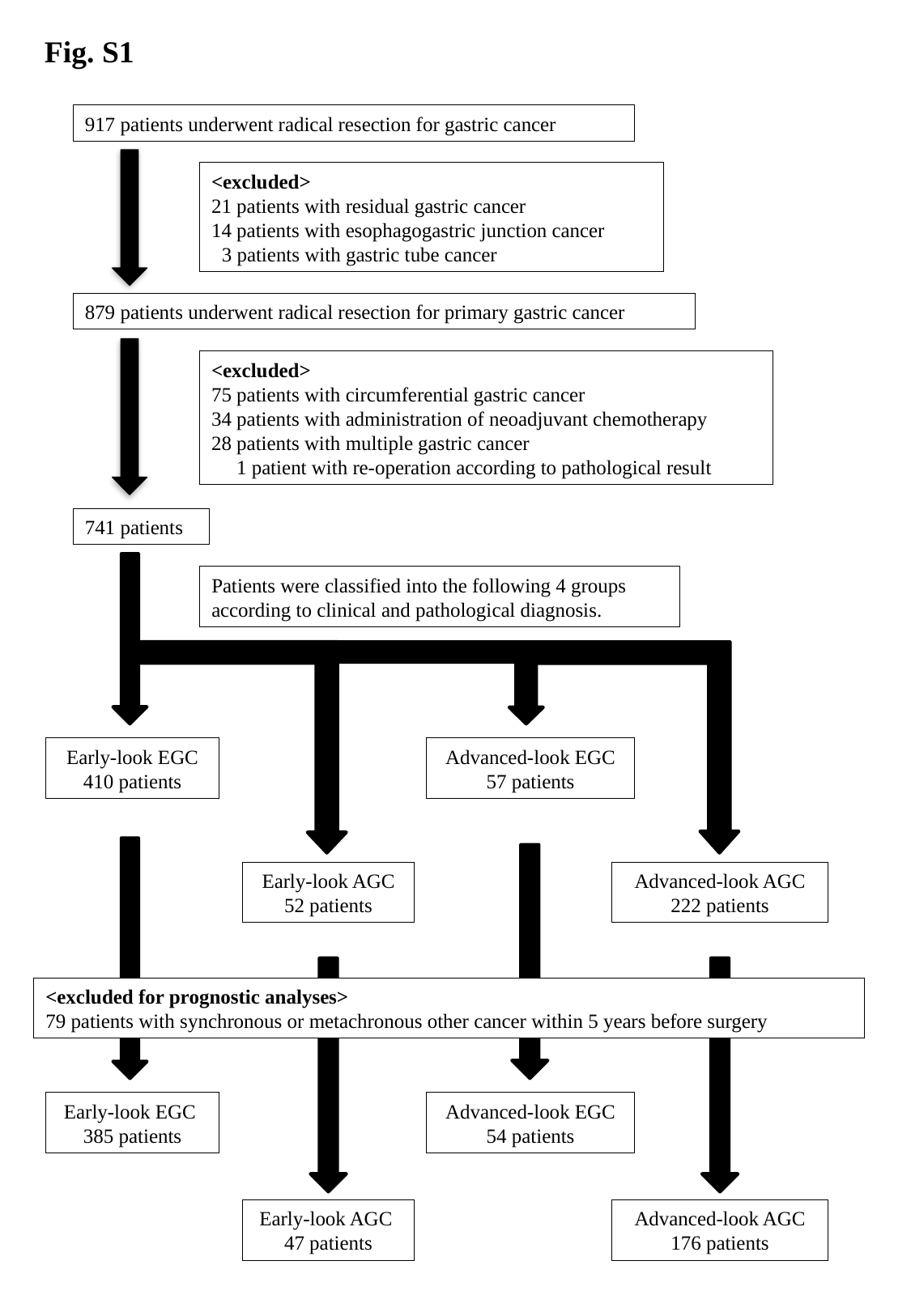

Fig. S1
917 patients underwent radical resection for gastric cancer
<excluded>
21 patients with residual gastric cancer
14 patients with esophagogastric junction cancer
 3 patients with gastric tube cancer
879 patients underwent radical resection for primary gastric cancer
<excluded>
75 patients with circumferential gastric cancer
34 patients with administration of neoadjuvant chemotherapy
28 patients with multiple gastric cancer
　1 patient with re-operation according to pathological result
741 patients
Patients were classified into the following 4 groups
according to clinical and pathological diagnosis.
Early-look EGC 410 patients
Advanced-look EGC
57 patients
Early-look AGC
52 patients
Advanced-look AGC
222 patients
<excluded for prognostic analyses>
79 patients with synchronous or metachronous other cancer within 5 years before surgery
Early-look EGC
385 patients
Advanced-look EGC
54 patients
Early-look AGC
47 patients
Advanced-look AGC
176 patients
