# Supplementary material for: Diagnostic accuracy of the gastric cancer T-category with respect to tumor localization
Source: Langenbecks Arch Surg. 2020 Aug 26;405(6):787–96. doi: 10.1007/s00423-020-01971-3 (PMC7471143; doi:10.1007/s00423-020-01971-3)
Supplement: Supplementary file 2 — Relation between clinical T-category and survival curve (OS and RFS) by each pStage. Kaplan–Meier curves for the RFS (left) and OS (right) rates of GC patients by each pStage. (PPTX 328 kb). [file 423_2020_1971_MOESM2_ESM.pptx]

## Slide 1
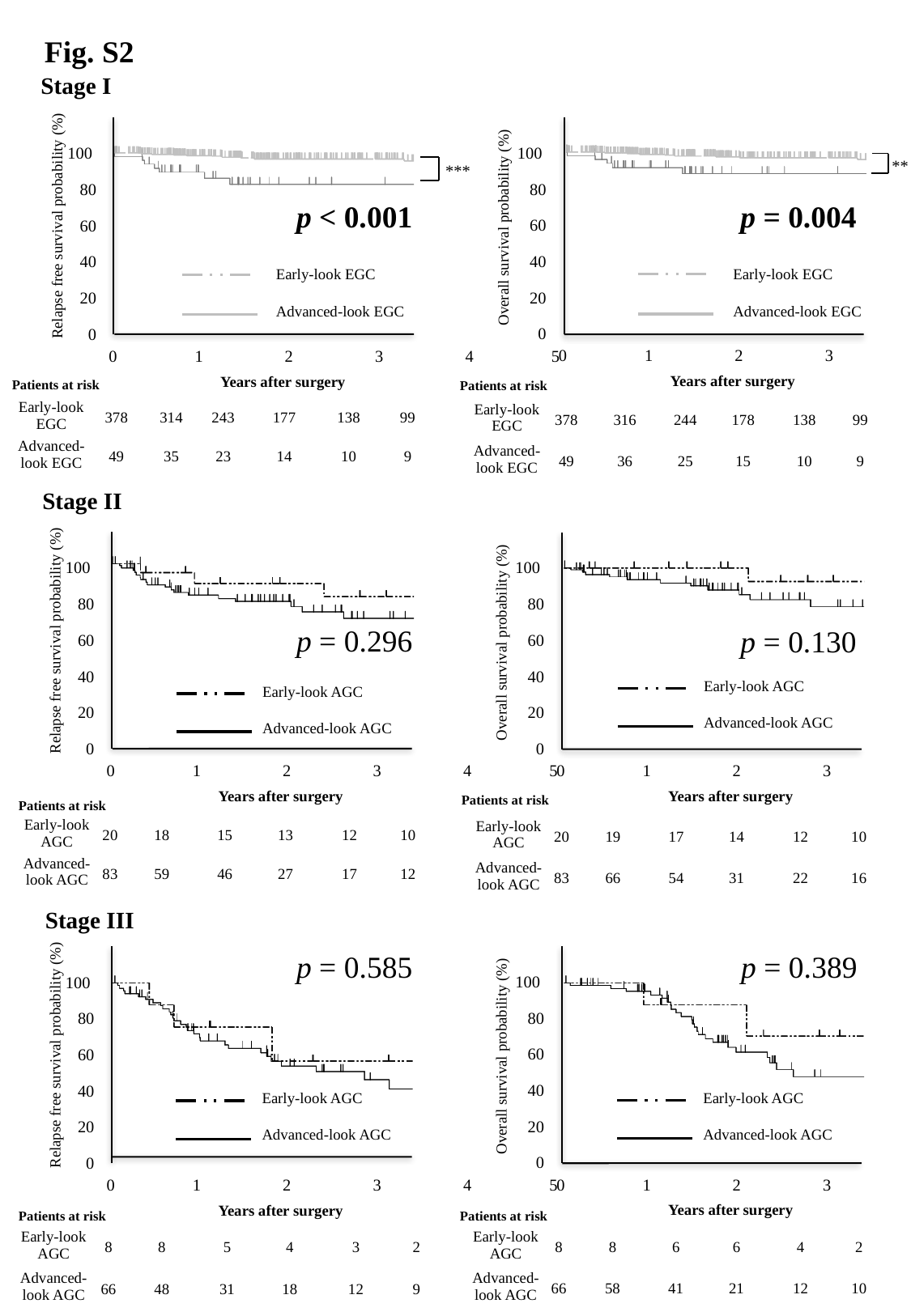

Fig. S2
Stage I
100
80
60
40
20
0
100
80
60
40
20
0
**
***
p = 0.004
p < 0.001
Relapse free survival probability (%)
Overall survival probability (%)
Early-look EGC
Advanced-look EGC
Early-look EGC
Advanced-look EGC
0　　　　 1 　　　　 2　 　　　 3　 　　　 4　　　　 5
0　　　　 1 　　　　 2　 　　　 3　 　　　 4　　　　 5
Years after surgery
Years after surgery
Patients at risk
Patients at risk
| Early-look EGC | 378 | 314 | 243 | 177 | 138 | 99 |
| --- | --- | --- | --- | --- | --- | --- |
| Advanced-look EGC | 49 | 35 | 23 | 14 | 10 | 9 |
| Early-look EGC | 378 | 316 | 244 | 178 | 138 | 99 |
| --- | --- | --- | --- | --- | --- | --- |
| Advanced-look EGC | 49 | 36 | 25 | 15 | 10 | 9 |
Stage II
100
80
60
40
20
0
100
80
60
40
20
0
p = 0.296
p = 0.130
Relapse free survival probability (%)
Overall survival probability (%)
Early-look AGC
Advanced-look AGC
Early-look AGC
Advanced-look AGC
0　　　　 1 　　　　 2　 　　　 3　 　　　 4　　　　 5
0　　　　 1 　　　　 2　 　　　 3　 　　　 4　　　　 5
Years after surgery
Years after surgery
Patients at risk
Patients at risk
| Early-look AGC | 20 | 19 | 17 | 14 | 12 | 10 |
| --- | --- | --- | --- | --- | --- | --- |
| Advanced-look AGC | 83 | 66 | 54 | 31 | 22 | 16 |
| Early-look AGC | 20 | 18 | 15 | 13 | 12 | 10 |
| --- | --- | --- | --- | --- | --- | --- |
| Advanced-look AGC | 83 | 59 | 46 | 27 | 17 | 12 |
Stage III
p = 0.389
p = 0.585
100
80
60
40
20
0
100
80
60
40
20
0
Relapse free survival probability (%)
Overall survival probability (%)
Early-look AGC
Advanced-look AGC
Early-look AGC
Advanced-look AGC
0　　　　 1 　　　　 2　 　　　 3　 　　　 4　　　　 5
0　　　　 1 　　　　 2　 　　　 3　 　　　 4　　　　 5
Years after surgery
Years after surgery
Patients at risk
Patients at risk
| Early-look AGC | 8 | 8 | 6 | 6 | 4 | 2 |
| --- | --- | --- | --- | --- | --- | --- |
| Advanced-look AGC | 66 | 58 | 41 | 21 | 12 | 10 |
| Early-look AGC | 8 | 8 | 5 | 4 | 3 | 2 |
| --- | --- | --- | --- | --- | --- | --- |
| Advanced-look AGC | 66 | 48 | 31 | 18 | 12 | 9 |
