# Supplementary material for: Diagnostic accuracy of the gastric cancer T-category with respect to tumor localization
Source: Langenbecks Arch Surg. 2020 Aug 26;405(6):787–96. doi: 10.1007/s00423-020-01971-3 (PMC7471143; doi:10.1007/s00423-020-01971-3)
Supplement: Supplementary file 3 — Survival curve by prognostic factors in patients with pEGC. Kaplan–Meier curves for the RFS rates of GC patients by prognostic factors in patients with pEGC. (PPTX 208 kb). [file 423_2020_1971_MOESM3_ESM.pptx]

## Slide 1
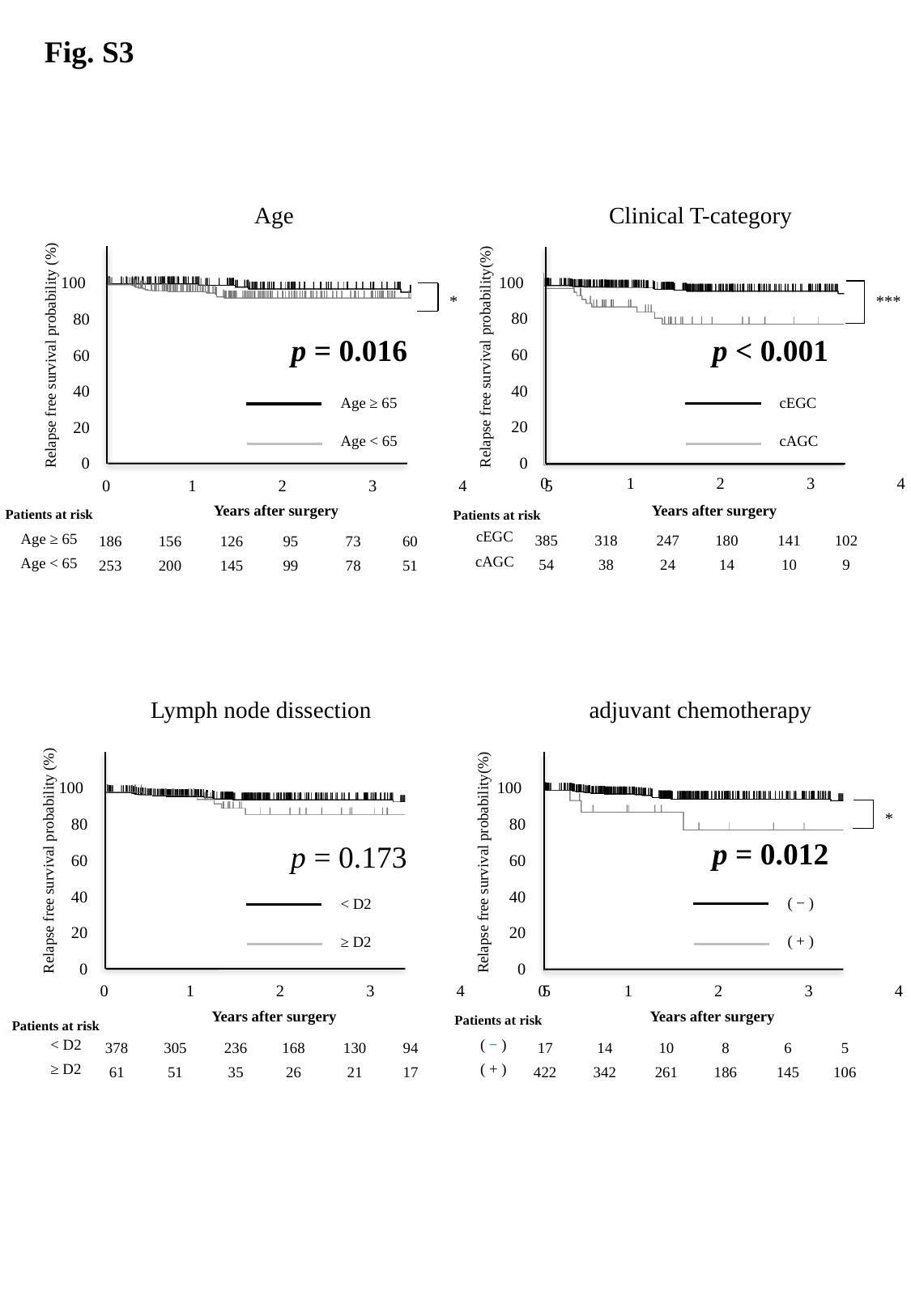

Fig. S3
Age
Clinical T-category
100
80
60
40
20
0
100
80
60
40
20
0
*
***
p = 0.016
p < 0.001
Relapse free survival probability (%)
Relapse free survival probability(%)
cEGC
cAGC
Age ≥ 65
Age < 65
0　　　　 1 　　　　 2　 　　　 3　 　　　 4　　　　 5
0　　　　 1 　　　　 2　 　　　 3　 　　　 4　　　　 5
Years after surgery
Years after surgery
Patients at risk
Patients at risk
| cEGC | 385 | 318 | 247 | 180 | 141 | 102 |
| --- | --- | --- | --- | --- | --- | --- |
| cAGC | 54 | 38 | 24 | 14 | 10 | 9 |
| Age ≥ 65 | 186 | 156 | 126 | 95 | 73 | 60 |
| --- | --- | --- | --- | --- | --- | --- |
| Age < 65 | 253 | 200 | 145 | 99 | 78 | 51 |
Lymph node dissection
adjuvant chemotherapy
100
80
60
40
20
0
100
80
60
40
20
0
*
p = 0.012
p = 0.173
Relapse free survival probability (%)
Relapse free survival probability(%)
( − )
( + )
< D2
≥ D2
0　　　　 1 　　　　 2　 　　　 3　 　　　 4　　　　 5
0　　　　 1 　　　　 2　 　　　 3　 　　　 4　　　　 5
Years after surgery
Years after surgery
Patients at risk
Patients at risk
| ( − ) | 17 | 14 | 10 | 8 | 6 | 5 |
| --- | --- | --- | --- | --- | --- | --- |
| ( + ) | 422 | 342 | 261 | 186 | 145 | 106 |
| < D2 | 378 | 305 | 236 | 168 | 130 | 94 |
| --- | --- | --- | --- | --- | --- | --- |
| ≥ D2 | 61 | 51 | 35 | 26 | 21 | 17 |
