# Supplementary material for: Diagnostic accuracy of the gastric cancer T-category with respect to tumor localization
Source: Langenbecks Arch Surg. 2020 Aug 26;405(6):787–96. doi: 10.1007/s00423-020-01971-3 (PMC7471143; doi:10.1007/s00423-020-01971-3)
Supplement: Supplementary file 4 [file 423_2020_1971_MOESM4_ESM.docx]

Table S1 Clinicopathological risk factors for diagnosing pEGC as cAGC

Variables		pEGC (%) n = 57	pAGC (%) n = 222	Univariate analysis	Multivariate analysis		
					Odds ratio	*p* value	95 % CI
Age							
	< 65	17 (29.8 %)	69 (31.1 %)	0.855			
	≥ 65	40 (70.2 %)	153 (68.9 %)				
Sex							
	M	38 (66.7 %)	161 (72.5 %)	0.383			
	F	19 (33.3 %)	61 (27.5 %)				
BMI							
	≥ 22	31 (54.4 %)	104 (46.8 %)	0.310			
	< 22	26 (45.6 %)	118 (53.2 %)				
Histopathology[b] (biopsy)							
	intestinal type	33 (58.9 %)	128 (60.1 %)	0.874			
	diffuse type	23 (41.1 %)	85 (39.9 %)				
	unknown	1	9				
Tumor localization							
	L	28 (49.1 %)	72 (32.4 %)	**0.019**	**2.18**	**0.021**	**1.13 - 4.24**
	other	29 (50.9 %)	150 (67.6 %)				
Tumor size (mm)							
	< 20	7 (12.3 %)	2 (0.9 %)	**< 0.001**	**7.92**	**0.016**	**1.48 - 42.31**
	≥ 20	50 (87.7 %)	220 (99.1 %)				
Macroscopic appearance							
	localized	20 (35.1 %)	87 (39.2 %)	0.570			
	diffuse	37 (64.9 %)	135 (60.8 %)				
Surgical approach							
	open	40 (70.2 %)	199 (89.6 %)	**< 0.001**	**2.46**	**0.027**	**1.11 - 5.43**
	laparoscopic	17 (29.8 %)	23 (10.4 %)				
Surgical procedure							
	TG	18 (32.1 %)	101 (46.5 %)	0.148			
	DG	38 (67.9 %)	116 (53.5 %)				
	Others	1	5				
Lymph node disection							
	< D2	17 (29.8 %)	52 (23.4 %)	0.318			
	≥ D2	40 (70.2 %)	170 (76.6 %)				
Adjuvant chemotherapy							
	(+)	5 (8.9 %)	103 (47.9 %)	**< 0.001**	**7.71**	**< 0.001**	**2.91 - 20.47**
	(−)	51 (91.1 %)	112 (52.1 %)				
	unknown	1	7				

a According to Lauren classification using most predominant histopathological finding.
cAGC, clinical advanced gastric cancer; pEGC, pathological early gastric cancer; pAGC, pathological advanced gastric cancer; HR, hazard ratio; BMI, body mass index; TG, total gastric cancer; DG, distal gastric cancer.

Table S2 Multivariate analysis for clinicopathologic characteristics affecting prognosis in patients with pEGC

Variables	RFS			OS	
	HR	95 % CI	*p* value	HR	95 % CI
Age ≥ 65 (vs. < 65)	**5.32**	**1.30 - 36.81**	**0.018**	**6.05**	**1.38 - 43.93**
Female (vs. male)	0.82	0.20 - 2.93	0.764	1.09	0.26 - 4.12
BMI < 22 (vs. ≥ 22)	0.88	0.27 - 2.77	0.835	1.76	0.56 - 5.50
Tumor size (mm) ≥ 20 (vs. < 20)	1.14	0.31 - 5.74	0.857	0.80	0.22 - 3.81
cAGC (vs. cEGC)	**8.06**	**1.49 - 45.24**	**0.016**	4.93	0.89 - 29.62
Macroscopic diffuse appearance (vs. localized appearance)	0.24	0.05 - 1.04	0.057	**0.19**	**0.04 - 0.92**
TG (vs. DG)	1.54	0.38 - 5.12	0.518	1.04	0.21 - 3.78
Lymph node dissection < D2 (vs. ≥ D2)	**7.43**	**1.44 - 46.12**	**0.016**	**7.91**	**1.30 - 62.49**
Histopathlogical intestinal type (vs. diffuse type)[a]	2.77	0.80 - 11.65	0.110	3.56	0.92 - 17.33
pN stage pN1-3 (vs. pN0)	2.96	0.73 - 10.99	0.126	3.48	0.76 - 14.57
Lymphatic invasion (vs. negative)	0.69	0.16 - 2.57	0.592	0.65	0.13 - 2.60
Venous invasion (vs. negative)	0.71	0.17 - 2.53	0.617	0.58	0.11 - 2.33
Adjuvant chemotherapy (vs. absence)	**9.71**	**1.58 - 52.45**	**0.016**	**11.91**	**1.78 - 72.33**

a According to Lauren classification using most predominant histopathological finding.

pEGC, pathological early gastric cancer; cAGC, clinical advanced gastric cancer; cEGC, clinical early gastric cancer; RFS, relapse free survival; OS, overall survival, HR, hazard ratio; CI, confident interval; BMI, body mass index, TG, total gastric cancer; DG, distal gastric cancer.
